# Supplementary material for: Identification of photoperiod-induced specific miRNAs in the adrenal glands of Sunite sheep (Ovis aries)
Source: Front Vet Sci. 2022 Jul 22;9:888207. doi: 10.3389/fvets.2022.888207 (PMC9354845; doi:10.3389/fvets.2022.888207)
Supplement: Supplementary file 1 [file Data_Sheet_1.ZIP › Supplementary file/miRDeep2 file.zip!/miRDeep2 file/Know_Structure/LP42A1/oar-mir-376d.pdf]

miRBase precursor : oar-mir-376d
Total read count : 239
oar-miR-376d read count : 234
remaining reads : 5

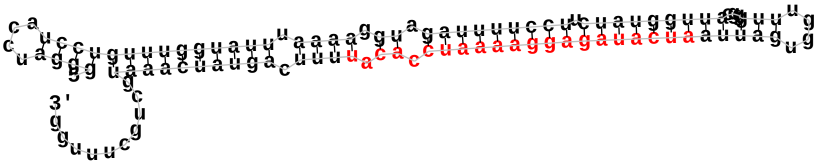

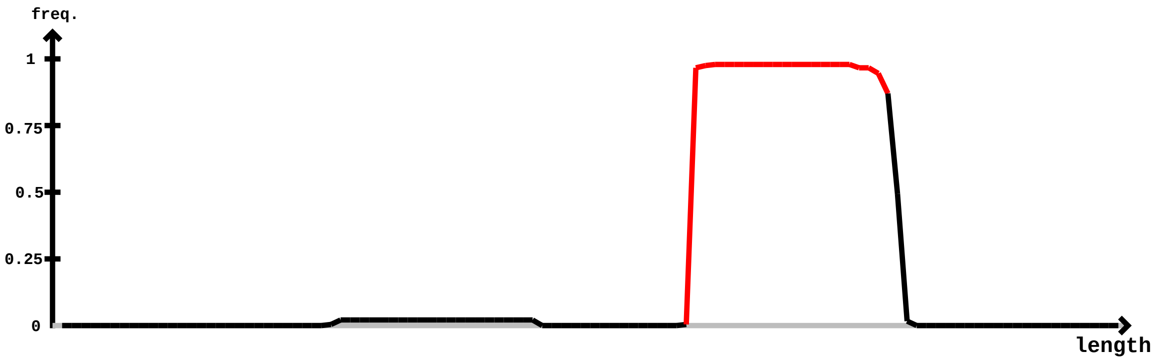

oar-miR-376d

```
5'- gggauccauccuguuugguauuuaaaagguagauuuuccuucuaugguuacggguuuggugauuaaucauagaggaaaauccacauuuucaguaucaaaugcugcuuugg   -3'    exp
    ((((....))))((((((((((.((((.((.(((((((((.(((((((((...(((....)))))))))))))))))))))).)).)))).))))))))))..........     reads       mm         sample
    ...........................gguagauuuuccuucuaugguu.............................................................     1           0          seq
    ............................guagauuuuccuucuaugguu.............................................................     4           0          seq
    ................................................................aaucauagaggaaaauccacau........................     1           0          seq
    .................................................................aucauagaggaaaaucc............................     2           0          seq
    .................................................................aucCuagaggaaaaucc............................     1           1          seq
    .................................................................aucauagaggaaaauccac..........................     5           0          seq
    .................................................................aucauagaggaaaauccaca.........................     18          0          seq
    .................................................................aucauagaggaaaauccacau........................     89          0          seq
    .................................................................aucauagaggaaaauccacauu.......................     112         0          seq
    .................................................................aucauagaggaaaauccacauuu......................     3           0          seq
    ..................................................................ucauagaggaaaauccacauu.......................     1           0          seq
    ..................................................................ucauagaggaaaauccacauuu......................     1           0          seq
    ...................................................................cauagaggaaaauccacauu.......................     1           0          seq
```
